# Triglyceride-glucose index and non-culprit coronary plaque characteristics assessed by optical coherence tomography in patients following acute coronary syndrome: A cross-sectional study

**DOI:** 10.3389/fcvm.2022.1019233

**Published:** 2022-10-12

**Authors:** Zi-Wei Zhao, Chi Liu, Qi Zhao, Ying-Kai Xu, Yu-Jing Cheng, Tie-Nan Sun, Yu-Jie Zhou

**Affiliations:** ^1^Beijing Key Laboratory of Precision Medicine of Coronary Atherosclerotic Disease, Department of Cardiology, Clinical Center for Coronary Heart Disease, Beijing Anzhen Hospital, Beijing Institute of Heart Lung and Blood Vessel Disease, Capital Medical University, Beijing, China; ^2^Department of Cardiology, Cardiovascular Center, Beijing Friendship Hospital, Capital Medical University, Beijing, China

**Keywords:** triglyceride-glucose index, optical coherence tomography, non-culprit coronary plaque, acute coronary syndrome, risk predictor

## Abstract

**Background:**

Triglyceride-glucose (TyG) index, a novel surrogate marker of insulin resistance, has been demonstrated to be significantly associated with cardiovascular disease. It remains indistinct regarding the association between TyG index and non-culprit coronary plaque characteristics in patients following acute coronary syndrome (ACS).

**Methods:**

The present study retrospectively recruited patients who were diagnosed with ACS and underwent non-culprit optical coherence tomography (OCT) examination. The study population was divided into 2 groups based on the median of TyG index, which was calculated as Ln [fasting triglyceride (TG) (mg/dL) × fasting blood glucose (FBG) (mg/dL)/2]. The non-culprit plaque characteristics were determined by interpreting OCT images in accordance with the standard of previous consensus.

**Results:**

110 patients (54.8 ± 12.1 years, 24.5% female) with 284 non-culprit plaques were included in the current analysis. TyG index was closely associated with high-risk plaque characteristics. Elevated TyG index was consistent to be an independent indicator for thin-cap fibroatheroma (TCFA) [odds ratio (OR) for per 1-unit increase 4.940, 95% confidence interval (CI) 1.652–14.767, P = 0.004; OR for taking lower median as reference 2.747, 95% CI 1.234–7.994, P = 0.011] and ruptured plaque (OR for per 1–unit increase 7.065, 95% CI 1.910–26.133, P = 0.003; OR for taking lower median as reference 4.407, 95% CI 1.208–16.047, P = 0.025) in fully adjusted model. The predictive value of TyG index for TCFA and ruptured plaque was moderate–to–high, with the area under the receiver operating characteristic curve (AUC) of 0.754 and 0.699 respectively. The addition of TyG index into a baseline model exhibited an incremental effect on the predictive value for TCFA, manifested as an increased AUC (0.681, 95% CI 0.570–0.793 vs. 0.782, 95% CI 0.688–0.877, P = 0.042), and significant continuous net reclassification improvement (0.346, 95% CI 0.235–0.458, P < 0.001) and integrated discrimination improvement (0.221, 95% CI 0.017–0.425, P = 0.034). TyG index failed to play an incremental effect on predicting ruptured plaque.

**Conclusion:**

TyG index, which is simply calculated from fasting TG and FBG, can be served as an important and independent risk predictor for high-risk non-culprit coronary plaques in patients following ACS.

## Background

Coronary artery disease (CAD) has become one of the most important chronic non-communicable diseases leading to death and disability, which brings heavy social and economic burden (1). Despite of the popularization and promotion for secondary preventive medications and revascularization therapies, patients with CAD remain to be at high risk of recurrent cardiovascular events, particularly for those who have ever experienced an acute coronary syndrome (ACS), the major pathogenesis of which has been recognized as thrombosis secondary to plaque rupture (2, 3). The close relationship between culprit plaque features and prognosis has been well established by previous studies (4). In clinical practice, the plaques in culprit lesions, indeed, are of the greatest concern and acquire the priority for intervention in patients with ACS. However, former study has also revealed that vulnerable plaques in non-culprit lesions are significantly associated with the subsequent ACS at the lesion level (5), indicating the important role of non-culprit plaque characteristics in prognostic prediction. Exploring novel risk factors with the aim of early identification of high-risk non-culprit plaques, therefore, is of great clinical significance for the prediction and prevention of recurrent cardiovascular events.

Insulin resistance (IR) has been recognized as one of the most significant risk factors associated with the development, progression, and prognosis of cardiovascular disease, independent of the glucometabolic status (6, 7). Triglyceride-glucose (TyG) index, a surrogate marker of IR being simple to calculate and easy to access from fasting triglyceride (TG) and fasting blood glucose (FBG), has been proposed and verified to be highly correlated to IR estimated by gold standard, the hyperinsulinaemic-euglycaemic clamp (8, 9). It has been demonstrated that higher TyG index can predict the incidence of cardiovascular risk factors including diabetes, prediabetes, and hypertension (10–12). More importantly, an increased level of TyG index has been also proved to play a pivotal role in the identification of individuals who are prone to cardiovascular disease and the prediction of adverse prognosis for patients with pre-existing cardiovascular disease (13–15).

At present, there is relatively inadequate evidence as regards the association between TyG index and non-culprit coronary plaque characteristics in patients who experienced ACS. The current study, therefore, was designed to explore the underlying relationship of TyG index with non-culprit plaque characteristics and phenotypes assessed by optical coherence tomography (OCT), a cross-sectional intracoronary imaging approach with high resolution providing the most detailed information about plaque morphology and microstructure, and to determine the potential of TyG index as a useful predictor for high-risk non-culprit plaques.

## Methods

### Study population

**Figure 1:**
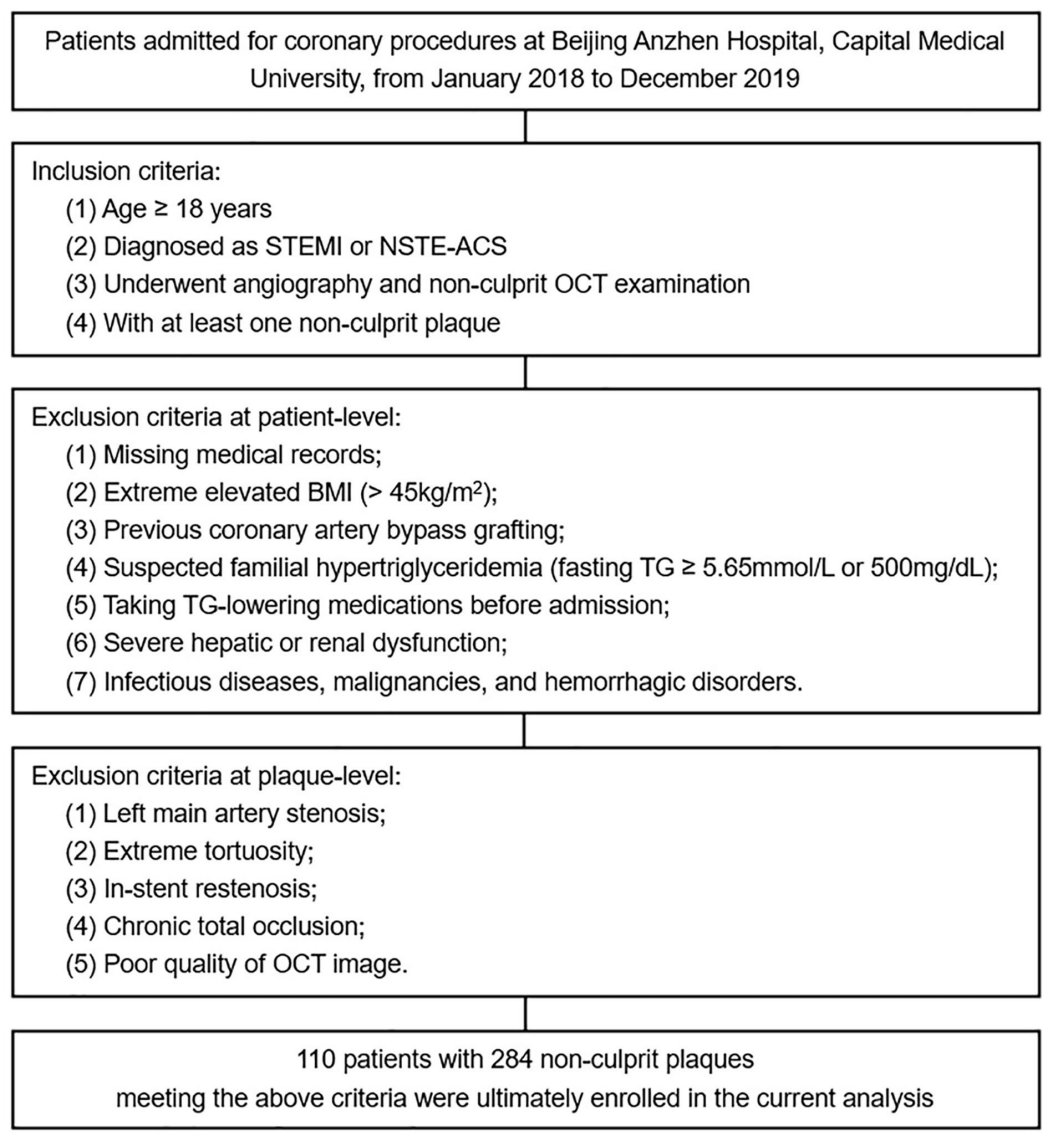
Flow diagram for the current study. STEMI, ST-segment elevation myocardial infarction; NSTE-ACS, non-ST-segment elevation acute coronary syndrome; OCT, optical coherence tomography; BMI, body mass index; TG, triglyceride.

As a single-center cross-sectional study, we retrospectively screened patients admitted for coronary procedures at Beijing Anzhen Hospital, Capital Medical University, from January 2018 to December 2019. The inclusion criteria were summarized as follows: (1) age ≥ 18 years; (2) diagnosed as ST-segment elevation myocardial infarction or non-ST-segment elevation ACS; (3) underwent angiography and non-culprit OCT examination; (4) with at least one non-culprit plaque. Patients with missing medical records, extreme elevated body mass index (BMI), previous coronary artery bypass grafting, etc., and plaques with left main artery stenosis, extreme tortuosity, in-stent restenosis, etc. were excluded (details shown in Figure 1).

The study was endorsed by the Clinical Research Ethics Committee of Beijing Anzhen Hospital, Capital Medical University. Consents were acquired from all enrolled patients for participating in the current analysis.

### Baseline data collection

Information concerning demographics, anthropometrics, past medical history, and laboratory examinations was extracted from the medical record. Then the obtained information was inputted into an established electronic database by two independent personnel who were ignorant of the study design.

BMI was calculated as weight (kg)/[height (m)]^2^. Patients who were still smoking at admission or had been abstinent for <1 year were regarded as current smoking. Patients with pre-existing or newly diagnosed hypertension and/or diabetes mellitus during hospitalization were considered to have hypertension and/or diabetes mellitus. Previous medical history of myocardial infarction and percutaneous coronary intervention was acquired from self-report and then verified by referring to relevant data. Laboratory parameters collected for the current analysis were all tested in the central laboratory of the hospital by using venous samples with fasting time over 12 h. The TyG index was calculated as previously described: Ln [fasting TG (mg/dL) × FBG (mg/dL)/2] (8).

### Angiographic and OCT-derived data interpretation

Coronary angiography and non-culprit OCT examination (The C7XR OCT system, St. Jude Medical, St. Paul, MN, USA) were performed by experienced cardiologists who were unaware of the study design. The angiographic and OCT-derived data were interpreted and recorded by two independent cardiologists who were blinded to the clinical and laboratory data of the study population. Disagreements encountered during the interpretation of angiographic and OCT images were settled by seeking help from another experienced cardiologist.

The cross-sectional OCT images were interpreted with 1 mm intervals. Non-culprit plaques were identified by OCT and defined as the presence of segments losing the normal 3-layered vessel structure in ≥ 3 consecutive cross-sectional images, excluding culprit plaques which were determined based on findings from angiogram, electrocardiogram, and/or echocardiogram. Segments with longitudinal distance of <5 mm or ≥ 5 mm were considered as single or independent plaques respectively.

**Figure 2:**
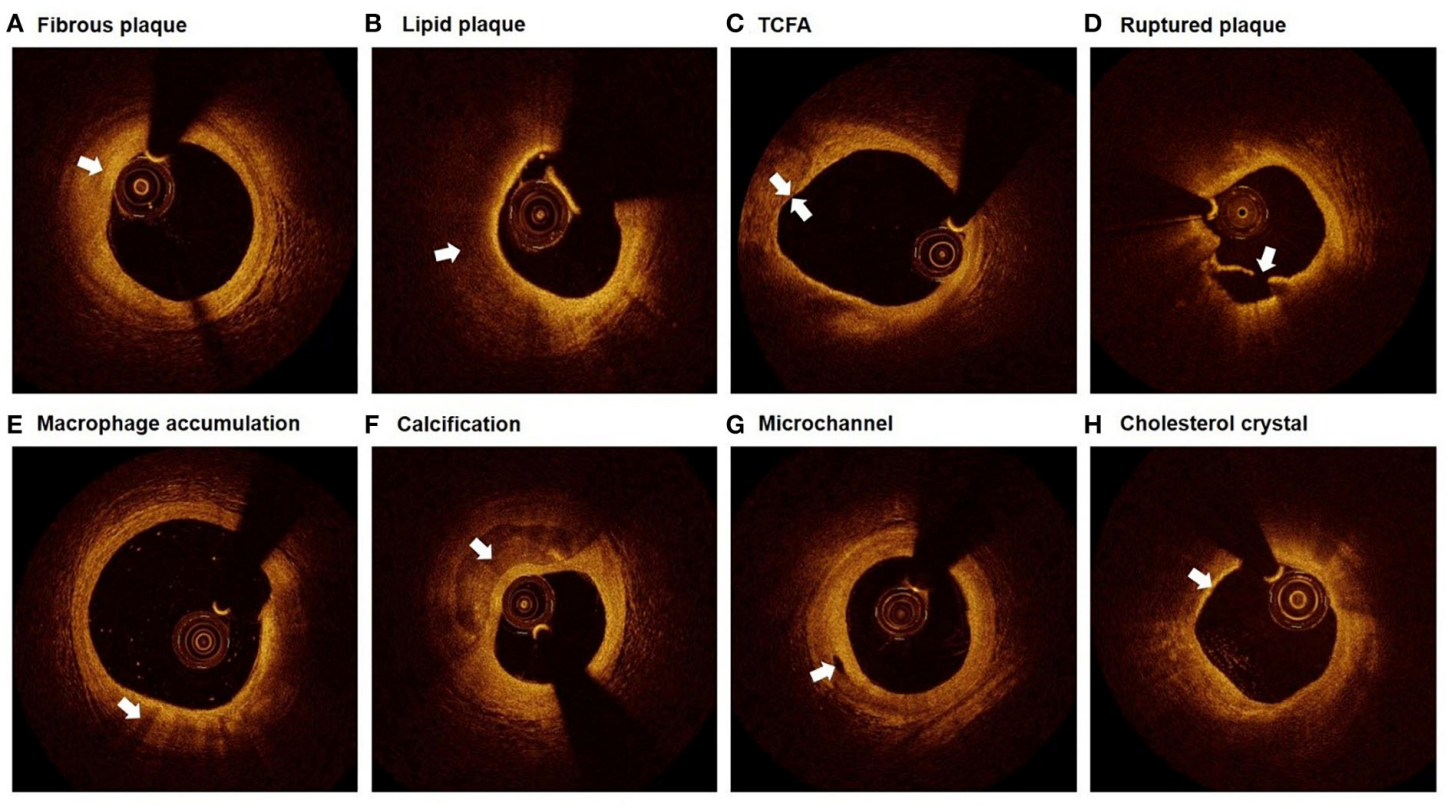
Representative plaque characteristics in OCT images. **(A)** Fibrous plaque: homogeneous signal and high backscattering; **(B)** Lipid plaque: poor signal, low backscattering, and diffuse border; **(C)** TCFA: lipid plaque with maximum lipid arc > 90° and FCT ≤ 65 μm; **(D)** Ruptured plaque: discontinuation of fibrous cap and the formation of cavity; **(E)** Macrophage accumulation: signal-rich, distinct, or confluent punctuate region with higher intensity than background speckle noise; **(F)** Calcification: low backscattering, poor or heterogeneous signal, and sharp border; **(G)** Microchannel: signal-poor tubular structure, present in ≥3 consecutive cross-sectional images, and not connected to the vessel lumen; **(H)** Cholesterol crystal: thin and linear structure with high signal and backscattering. TCFA, thin-cap fibroatheroma.

Plaque characteristics were defined by referring to relevant consensus standards for OCT (16). Each plaque was categorized as fibrous plaque or lipid plaque. Fibrous plaque was characterized as homogeneous signal and high backscattering (Figure 2A), while lipid plaque was characterized as poor signal, low backscattering, and diffuse border (Figure 2B). When a lipid plaque was confirmed, fibrous cap thickness (FCT), lipid arc, lipid core length, and lipid index were determined subsequently. FCT was defined as the mean value of 3 measurements at the thinnest part of the fibrous cap (a signal-rich homogenous layer overlying the lipid plaque). Lipid arc was measured through the entire length of each lipid plaque, the maximum and mean value of which were then determined. Lipid core length was measured as the longitudinal span of the entire lipid core for each lipid plaque. Lipid index was calculated as lipid core length × mean lipid arc. Thin-cap fibroatheroma (TCFA) was defined as the lipid plaque with maximum lipid arc > 90° and FCT ≤ 65μm (Figure 2C). Ruptured plaque was characterized as the discontinuation of fibrous cap and the formation of cavity within the plaque (Figure 2D). Macrophage accumulation was manifested as signal-rich, distinct, or confluent punctuate region with higher intensity than background speckle noise (Figure 2E). Calcification was presented as the region with low backscattering, poor or heterogeneous signal, and sharp border inside the plaque (Figure 2F). Calcification arc was measured through the entire cross-sectional images and calcification length was acquired by interpreting the longitudinal view. Calcification with arc ≤ 90° and length of 1–4mm were classified as spotty calcification. Microchannel was identified as signal-poor tubular structure (50–300 μm) that presented in ≥3 consecutive cross-sectional images and not connected to the vessel lumen (Figure 2G). Cholesterol crystal was defined as the thin and linear structure within the plaque exhibited as high signal and backscattering (Figure 2H). Thrombus was manifested as the mass locating on the luminal surface of the plaque or floating within the lumen, the former of which was brought into the current analysis.

### Statistical analysis

Continuous parameters with normal distribution were displayed as mean ± standard deviation and compared by *T* test. While for continuous variates with non-normal distribution, median with interquartile range was used for description and Mann-Whitney *U* test was performed to detect differences. Nominal parameters were described as absolute values with percentages and compared by Pearson chi-square test, chi-square test with continuity correction, or Fisher's exact test correspondingly. Correlation between TyG index and lipid plaque characteristics was evaluated by scatter plot and linear regression analysis. Univariate and multivariate logistic regression analyses were performed to evaluate the value of TyG index in the prediction of TCFA and ruptured plaque. Three multivariate models were established to investigate the independent role of TyG index in predicting TCFA and ruptured plaque, variates of which were selected with reference to univariate analysis and clinical experience. The odds ratio (OR) and 95% confidence interval (CI) were examined by taking TyG index as continuous (per 1-unit increase) and nominal (lower median as reference) variate respectively. The predictive value of TyG index for TCFA and ruptured plaque was further evaluated by receiver operating characteristic (ROC) analysis, basing on which the area under the ROC curve (AUC) and optimal cut-off value with sensitivity, specificity, positive predictive value (PPV), and negative predictive value (NPV) were determined. Moreover, to assess the improvement in predictive value after adding TyG index into the model of classical risk factors, AUCs for respective models were evaluated and compared by Z test. Meanwhile, continuous net reclassification improvement (NRI) and integrated discrimination improvement (IDI) were also calculated to further assess the incremental effect of TyG index on the predictive value.

Statistical analyses were performed with SPSS 26.0 (IBM, Armonk, New York, USA), MedCalc 19.1 (Ostend, Belgium), and R Programming Language 3.6.3. A two-side *P* < 0.05 was considered statistically significant.

## Results

### Baseline characteristics of the study population

In total, 110 patients with 284 non-culprit plaques who met the enrollment criteria were ultimately included in this study. The mean age of the study population was 54.8 ± 12.1 years, and 24.5% of them were female. Patients were divided into two groups based on the median of TyG index (median: 8.92), with 55 patients in each group. As manifested in Table 1, patients with higher median of TyG index, in comparison with those with lower median, exhibited higher levels of TG, uric acid, FBG, and glycosylated hemoglobin A1c. While the level of high-density lipoprotein cholesterol decreased significantly with the increasing median of TyG index.

**Table 1:** Baseline characteristics of the study population.

	**All patients (*n* = 110)**	**Patients with lower TyG index (*n* = 55)**	**Patients with higher TyG index (n = 55)**	**P value**
Age, years	54.8 ± 12.1	55.2 ± 11.3	54.5 ± 13.0	0.785
Gender, female, n (%)	27 (24.5)	14 (25.5)	13 (23.6)	0.825
BMI, kg/m^2^	25.4 ± 3.2	24.8 ± 3.0	26.0 ± 3.3	0.051
Current smoking, n (%)	57 (51.8)	29 (52.7)	28 (50.9)	0.849
Hypertension, n (%)	66 (60.0)	33 (60.0)	33 (60.0)	1.000
Diabetes mellitus, n (%)	33 (30)	12 (21.8)	21 (38.2)	0.061
Previous MI, n (%)	18 (16.4)	9 (16.4)	9 (16.4)	1.000
Previous PCI, n (%)	18 (16.4)	6 (10.9)	12 (21.8)	0.122
Diagnosis, n (%)				0.768
STEMI	13 (11.8)	7 (12.7)	6 (10.9)	
NSTE-ACS	97 (88.2)	48 (87.3)	49 (89.1)	
Laboratory data				
TG, mmol/L	1.6 (1.1, 2.5)	1.1 (0.9, 1.3)	2.5 (1.9, 2.9)	< 0.001
TC, mmol/L	3.9 (3.3, 4.5)	3.7 (3.2, 4.4)	4.1 (3.6, 4.6)	0.057
LDL-C, mmol/L	2.2 (1.8, 2.7)	2.1 (1.7, 2.7)	2.3 (1.9, 2.9)	0.193
HDL-C, mmol/L	1.0 (0.8, 1.2)	1.1 (0.9, 1.4)	0.9 (0.8, 1.1)	< 0.001
hs-CRP, mg/L	1.1 (0.5, 2.8)	0.7 (0.4, 2.6)	1.5 (0.6, 3.1)	0.053
Creatinine, μmol/L	71.3 ± 13.7	69.2 ± 13.0	73.3 ± 14.1	0.111
Uric acid, μmol/L	346.6 ± 94.1	320.3 ± 84.9	372.9 ± 96.3	0.003
FBG, mmol/L	5.9 (5.3, 6.4)	5.6 (5.2, 6.0)	6.1 (5.7, 7.1)	< 0.001
HbA1c, %	5.8 (5.5, 6.4)	5.6 (5.4, 6.0)	6.0 (5.7, 6.7)	0.001
TyG index	8.9 ± 0.6	8.5 ± 0.3	9.4 ± 0.3	< 0.001

BMI, body mass index; MI, myocardial infarction; PCI, percutaneous coronary intervention; STEMI, ST-segment elevation myocardial infarction; NSTE-ACS, non-ST-segment elevation acute coronary syndrome; TG, triglyceride; TC, total cholesterol; LDL-C, low-density lipoprotein cholesterol; HDL-C, high-density lipoprotein cholesterol; hs-CRP, high-sensitivity C-reactive protein; FBG, fasting blood glucose; HbA1c, glycosylated hemoglobin A1c; TyG, triglyceride-glucose.

### TyG index is closely associated with nun-culprit plaque characteristics

As shown in Table 2, when evaluating the association of TyG index with OCT-derived plaque characteristics at patient-level, the results showed that patients with higher TyG index exhibited to have more non-culprit plaques [3.0 (2.0, 4.0) vs. 2.0 (1.0, 3.0), *P* = 0.036]. Moreover, the proportions of lipid plaque, TCFA, ruptured plaque, and macrophage accumulation were significantly higher in patients with higher TyG index.

**Table 2:** Patient-level angiographic and OCT analysis of non-culprit plaques comparing lower vs. higher TyG index.

	**All patients (*n* = 110)**	**Patients with lower TyG index (*n* = 55)**	**Patients with higher TyG index (*n* = 55)**	**P value**
Number of total non-culprit plaques	2.0 (2.0, 3.0)	2.0 (1.0, 3.0)	3.0 (2.0, 4.0)	0.036
Non-culprit plaque territory				
LAD	75 (68.2)	36 (65.5)	39 (70.9)	0.539
LCX	12 (10.9)	7 (12.7)	5 (9.1)	0.541
RCA	32 (29.1)	17 (30.9)	15 (27.3)	0.675
Fibrous plaque, n (%)	101 (91.8)	51 (92.7)	50 (90.9)	0.728
Lipid plaque, n (%)	74 (67.3)	31 (56.4)	43 (78.2)	0.015
TCFA, n (%)	32 (29.1)	9 (16.4)	23 (41.8)	0.003
Ruptured plaque, n (%)	22 (20.0)	6 (10.9)	16 (29.1)	0.017
Macrophage accumulation, n (%)	75 (68.2)	30 (54.5)	45 (81.8)	0.002
Calcification, n (%)	56 (50.9)	24 (43.6)	32 (58.2)	0.127
Spotty calcification, n (%)	47 (42.7)	21 (38.2)	26 (47.3)	0.335
Microchannel, n (%)	58 (52.7)	27 (49.1)	31 (56.4)	0.445
Cholesterol crystal, n (%)	65 (59.1)	28 (50.9)	37 (67.3)	0.081
Thrombus, n (%)	15 (13.6)	4 (7.3)	11 (20.0)	0.096

LAD, left anterior descending artery; LCX, left circumflex artery; RCA, right coronary artery; TCFA, thin-cap fibroatheroma; TyG, triglyceride-glucose.

**Figure 3:**
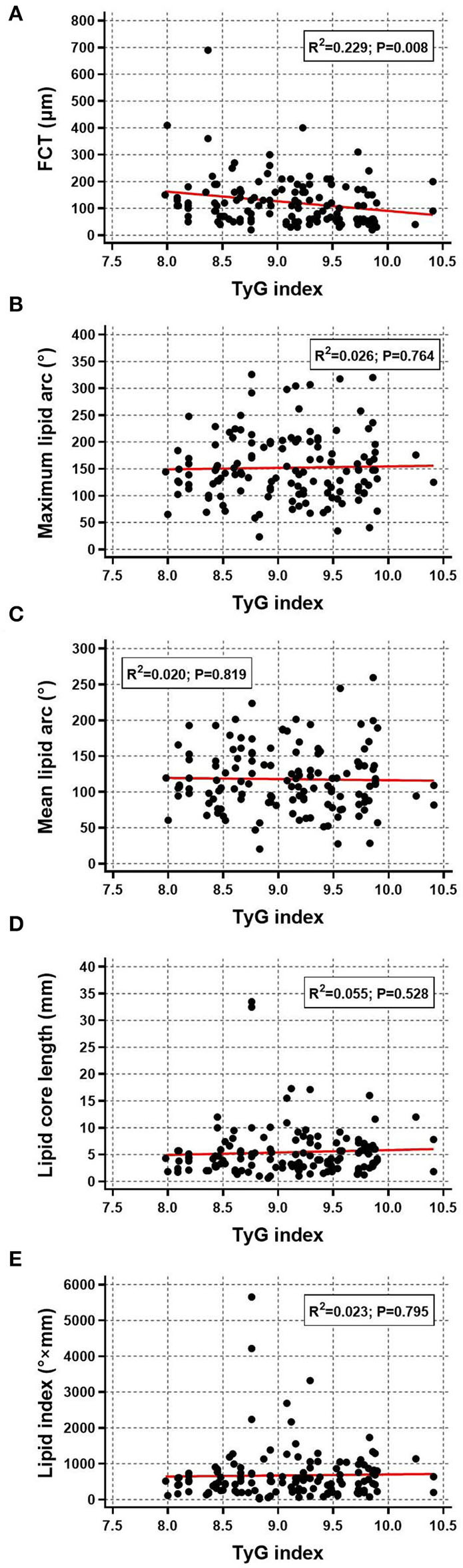
Correlation between TyG index and lipid plaque characteristics. Scatter plot and linear regression analysis were performed for TyG index and lipid plaque characteristics including **(A)** FCT, **(B)** maximum lipid arc, **(C)** mean lipid arc, **(D)** lipid core length, and **(E)** lipid index. FCT, fibrous cap thickness; TyG, triglyceride-glucose.

The relationship between TyG index and OCT-derived plaque characteristics was also assessed at plaque-level. Among the total 284 non-culprit plaques, those in higher TyG index group were more prone to be lipid plaque, TCFA, and ruptured plaque, and more likely to have macrophage accumulation, calcification, and microchannel (Table 3). Furthermore, as for lipid plaque characteristics, scatter plot and linear regression analysis were performed to investigate the correlation between TyG index and lipid plaque-related parameters, results of which revealed that there was a significant linear correlation between TyG index and FCT (R^2^ = 0.229, *P* = 0.008) (Figure 3A). The correlation of TyG index with maximum lipid arc, mean lipid arc, lipid core length, and lipid index, however, was not significant (all *P* > 0.05) (Figures 3B–E).

**Table 3:** Plaque-level angiographic and OCT analysis of non-culprit plaques comparing lower vs. higher TyG index.

	**All plaques (*n* = 284)**	**Plaques with lower TyG index (*n* = 132)**	**Plaques with higher TyG index (*n* = 152)**	**P value**
Non-culprit plaque territory				0.332
LAD	166 (58.5)	71 (53.8)	95 (62.5)	
LCX	29 (10.2)	15 (11.4)	14 (9.2)	
RCA	89 (31.3)	46 (34.8)	43 (28.3)	
Fibrous plaque, n (%)	151 (53.2)	80 (60.6)	71 (46.7)	0.019
Lipid plaque, n (%)	133 (46.8)	52 (39.4)	81 (53.3)	0.019
TCFA, n (%)	41 (14.4)	10 (7.6)	31 (20.4)	0.002
Ruptured plaque, n (%)	24 (8.5)	6 (4.5)	18 (11.8)	0.027
Macrophage accumulation, n (%)	133 (46.8)	46 (34.8)	87 (57.2)	< 0.001
Calcification, n (%)	104 (36.6)	40 (30.3)	64 (42.1)	0.039
Spotty calcification, n (%)	71 (25.0)	28 (21.2)	43 (28.3)	0.170
Microchannel, n (%)	92 (32.4)	35 (26.5)	57 (37.5)	0.049
Cholesterol crystal, n (%)	79 (27.8)	31 (23.5)	48 (31.6)	0.129
Thrombus, n (%)	15 (5.3)	4 (3.0)	11 (7.2)	0.189

LAD, left anterior descending artery; LCX, left circumflex artery; RCA, right coronary artery; TCFA, thin-cap fibroatheroma; TyG triglyceride-glucose.

### TyG index as an independent indicator of non-culprit TCFA and ruptured plaque

As mentioned above, the prevalence of TCFA and ruptured plaque went up as TyG index increased. Further univariate and multivariate logistic regression analyses were employed to confirm the association between TyG index and above two types of high-risk plaques. In univariate logistic regression analysis, elevated TyG index was consistent to be a significant indicator for TCFA (OR for per 1-unit increase 5.885, 95% CI 2.415–14.340, P < 0.001; OR for taking lower median as reference 3.674, 95% CI 1.504–8.972, *P* = 0.004) and ruptured plaque (OR for per 1–unit increase 3.905, 95% CI 1.566–9.736, *P* = 0.003; OR for taking lower median as reference 3.350, 95% CI 1.198–9.368, *P* = 0.021).

After taking the results from univariate analysis (Supplementary Table S1) and clinical experience into consideration, three multivariate models were established to investigate the independent association of TyG index with TCFA and ruptured plaque. The results revealed that a 1-unit increase of TyG index was independently and robustly associated with an increased risk of TCFA (Model 1: OR 5.838, 95% CI 2.353–14.485, *P* < 0.001; Model 2: OR 5.971, 95% CI 2.241–15.909, *P* < 0.001; Model 3: OR 4.940, 95% CI 1.652–14.767, *P* = 0.004) and ruptured plaque (Model 1: OR 5.349, 95% CI 1.883–15.197, *P* = 0.002; Model 2: OR 7.770, 95% CI 2.236–27.003, *P* = 0.001; Model 3: OR 7.065, 95% CI 1.910–26.133, *P* = 0.003). The independent association between TyG index and TCFA and ruptured plaque persisted when taking TyG index as a nominal variate (details shown in Table 4).

**Table 4:** Univariate and multivariate analysis evaluating the association of TyG index with non-culprit TCFA and ruptured plaque.

	**TCFA**			**Ruptured plaque**		
	**OR**	**95% CI**	**P value**	**OR**	**95% CI**	**P value**
Crude model						
TyG index, per 1-unit increase	5.885	2.415–14.340	< 0.001	3.905	1.566–9.736	0.003
TyG index, lower median as reference	3.674	1.504–8.972	0.004	3.350	1.198–9.368	0.021
Model 1						
TyG index, per 1-unit increase	5.838	2.353–14.485	< 0.001	5.349	1.883–15.197	0.002
TyG index, lower median as reference	3.981	1.577–10.050	0.003	3.647	1.253–10.614	0.018
Model 2						
TyG index, per 1-unit increase	5.971	2.241–15.909	< 0.001	7.770	2.236–27.003	0.001
TyG index, lower median as reference	3.718	1.406–9.832	0.008	5.255	1.505–18.348	0.009
Model 3						
TyG index, per 1-unit increase	4.940	1.652–14.767	0.004	7.065	1.910–26.133	0.003
TyG index, lower median as reference	2.747	1.234–7.994	0.011	4.407	1.208–16.047	0.025

Model 1: adjusted for age, gender, BMI, and current smoking.

Model 2: adjusted for Model 1 plus hypertension, diabetes mellitus, previous MI, previous PCI, and diagnosis.

Model 3: adjusted for Model 2 plus LDL-C, and HDL-C.

TyG, triglyceride-glucose; TCFA, thin-cap fibroatheroma; OR, odds ratio; CI, confidence interval.

### Predictive performance of TyG index for non-culprit TCFA and ruptured plaque

**Figure 4:**
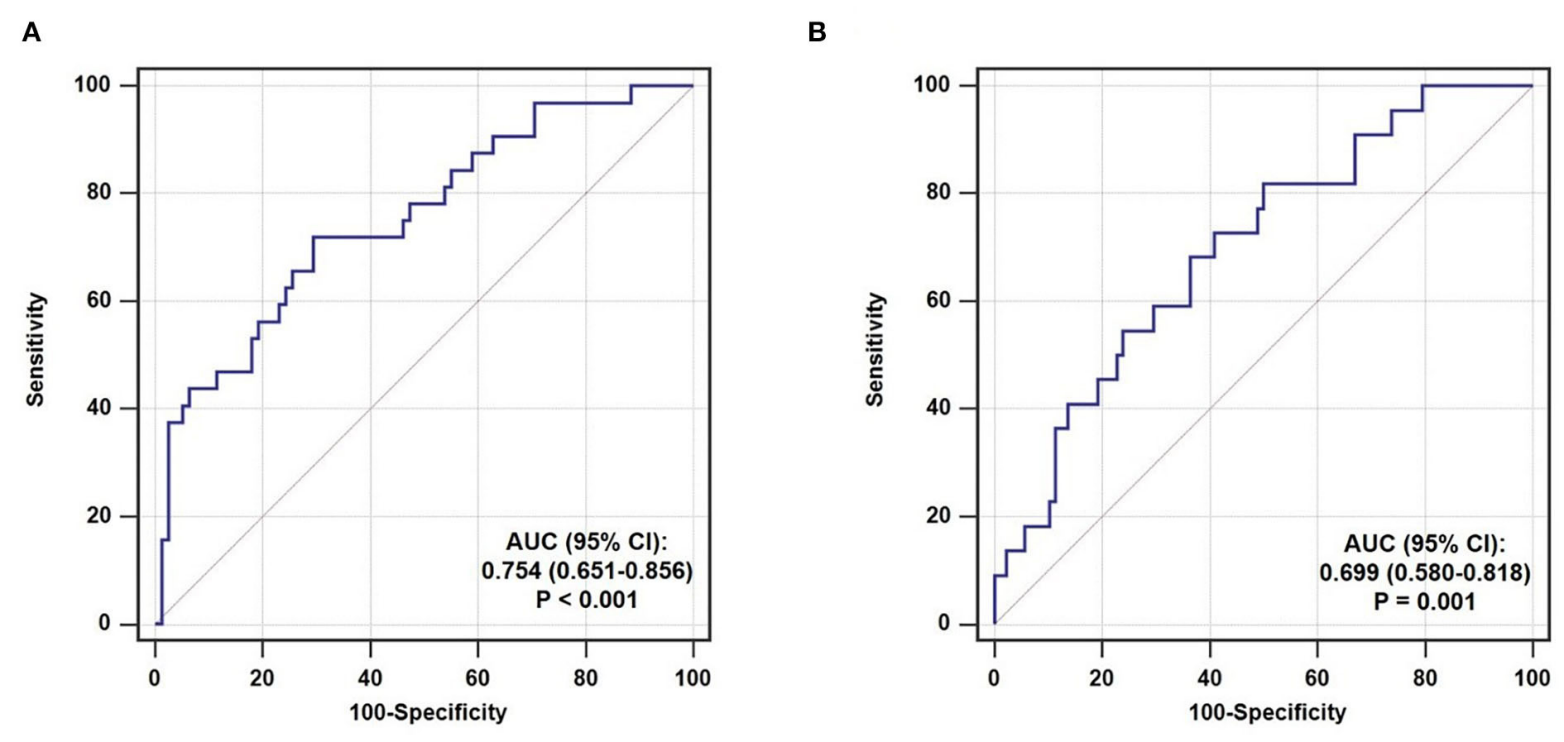
ROC curve analysis evaluating the predictive ability of TyG index for non-culprit **(A)** TCFA and **(B)** ruptured plaque. AUC, area under the ROC curve; CI, confidence interval.

The predictive performance of TyG index for TCFA and ruptured plaque was evaluated by ROC curve analysis. As shown in Figure 4A, TyG index displayed a moderate-to-high strength in the prediction of TCFA, with an AUC of 0.754 (95% CI 0.651–0.856, *P* < 0.001). The optimal cut-off value was 9.07, under which the sensitivity, specificity, PPV, and NPV were 71.9, 70.5, 50.0%, and 85.9% respectively (Table 5). The predictive value of TyG index for ruptured plaque, however, was mild, with a relatively lower AUC (0.699, 95% CI 0.580–0.818, *P* = 0.001), and at the optimal cut-off value of 8.75, the sensitivity, specificity, PPV, and NPV were 81.8, 50.0, 29.0, and 91.7% separately (Figure 4B, Table 5).

**Table 5:** Predictive performance of TyG index for non-culprit TCFA and ruptured plaque.

	**TCFA**	**Ruptured plaque**
AUC (95% CI)	0.754 (0.651–0.856)	0.699 (0.580–0.818)
Cut-off value	9.07	8.75
Sensitivity, % (95% CI)	71.9 (53.3–86.3)	81.8 (59.7–94.8)
Specificity, % (95% CI)	70.5 (59.1–80.3)	50.0 (39.1–60.9)
PPV, % (95% CI)	50.0 (40.0–60.0)	29.0 (23.5–35.3)
NPV, % (95% CI)	85.9 (77.5–91.5)	91.7 (81.6–96.5)

AUC, area under the ROC curve; CI, confidence interval; PPV, positive predictive value; NPV, negative predictive value; TCFA, thin-cap fibroatheroma.

### Incremental effect of TyG index on the prediction of non-culprit TCFA and ruptured plaque

**Figure 5:**
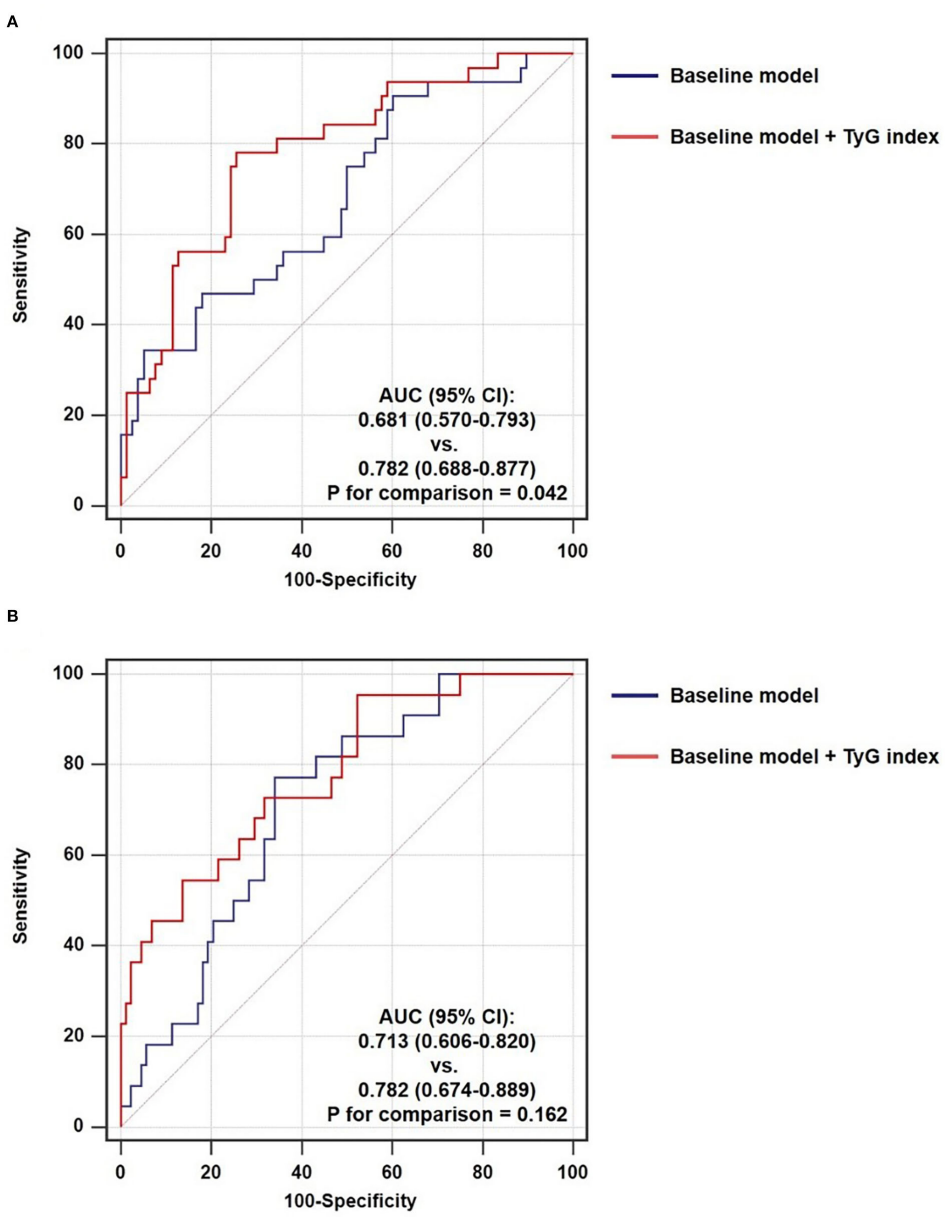
Incremental ability of TyG index on the prediction of non-culprit **(A)** TCFA and **(B)** ruptured plaque beyond a baseline model. AUC, area under the ROC curve; CI, confidence interval; TyG, triglyceride-glucose.

After introducing TyG index into a baseline model, which consisted of classical risk factors including age, gender, BMI, current smoking, hypertension, diabetes mellitus, and low-density lipoprotein cholesterol, there was a significant incremental effect on the predictive value for TCFA, manifested as an increased AUC (0.681, 95% CI 0.570–0.793 vs. 0.782, 95% CI 0.688–0.877, *P* = 0.042) (Figure 5A). In addition, the continuous NRI (0.346, 95% CI 0.235–0.458, *P* < 0.001) and IDI (0.221, 95% CI 0.017–0.425, *P* = 0.034) were also significant (Table 6). Nevertheless, the addition of TyG index to the same baseline model failed to contribute to a significant incremental effect on the predictive value for ruptured plaque (AUC: 0.713, 95% CI 0.606–0.820 vs. 0.782, 95% CI 0.674–0.889, *P* = 0.162; continuous NRI: 0.000, 95% CI −0.191–0.191, *P* > 0.999; IDI: 0.000, 95% CI −0.194–0.194, *P* > 0.999) (Figure 5B, Table 6).

**Table 6:** Incremental ability of TyG index on the predictive performance for non-culprit TCFA and ruptured plaque beyond classical risk factors.

	**AUC**			**Continuous NRI**		**IDI**	
	**Est. (95% CI)**	**ΔEst**.	**P value**	**Est. (95% CI)**	**P value**	**Est. (95% CI)**	**P value**
TCFA							
Baseline model^a^	0.681 (0.570–0.793)	–	–	–	–	–	–
+ TyG index	0.782 (0.688–0.877)	0.101	0.042	0.346 (0.235–0.458)	< 0.001	0.221 (0.017–0.425)	0.034
Ruptured plaque							
Baseline model^a^	0.713 (0.606–0.820)	–	–				
+ TyG index	0.782 (0.674–0.889)	0.069	0.162	0.000 (−0.191–0.191)	> 0.999	0.000 (−0.194–0.194)	> 0.999

TCFA, thin-cap fibroatheroma; TyG, triglyceride-glucose; AUC, area under the ROC curve; NRI, net reclassification improvement; IDI, integrated discrimination improvement; CI, confidence interval.

^a^The baseline model incorporates age, gender, BMI, current smoking, hypertension, diabetes mellitus, and LDL-C.

## Discussion

To our knowledge, the present study is the first to investigate the association between IR evaluated by TyG index and non-culprit coronary plaque characteristics assessed by OCT in patients following ACS. The major findings are as follows: (1) there was a close association between TyG index and high-risk plaque characteristics; (2) TyG index remained to be independently and robustly associated with high-risk plaques including TCFA and ruptured plaque with the adjustment of various multivariate models, despite being taken as a continuous or nominal variate; (3) the predictive value of TyG index for TCFA and ruptured plaque was moderate-to-high; (4) after being introduced into a baseline model, TyG index exhibited a significant incremental effect on the predictive value for TCFA, but not for ruptured plaque.

### Methods for estimation of IR and the superiority of TyG index

IR has been comprehensively demonstrated to be significantly associated with the prevalence, development, and prognosis of cardiovascular disease (6, 7). Thus, it appeals to great requirements on quantification of IR levels for individuals susceptible to or with pre-existing cardiovascular disease, with the aim to provide more information on risk prediction and stratification. Since the gold standard method for assessing IR, the hyperinsulinaemic-euglycaemic clamp, is complex, time-consuming, and expensive to operate, various surrogate markers of IR have been proposed for estimating the level of IR alternatively and verified to be highly correlated to the hyperinsulinaemic-euglycaemic clamp (17), which makes it possible to evaluate the extent of IR more quickly and feasibly, while maintaining accuracy.

Homeostasis model assessment of insulin resistance (HOMA-IR), which is calculated from FBG and fasting insulin, is used to being considered as the most recognized surrogate marker of IR (17). However, the examination of fasting insulin is not routinely performed in daily clinical processes, even for patients having experienced diabetes mellitus. In addition, although healthy subjects may manifest as a wide range of IR levels, fasting insulin values maintain within narrow ranges, suggesting that small but clinically relevant changes in IR may be masked by indicators calculated from fasting insulin that appear normal. Furthermore, there is a lack of consistent standards for the measurement of insulin, which results in nonnegligible heterogeneity across laboratories from different centers, especially for non-diabetic patients whose insulin concentration keeps at low levels. The limitations described above, therefore, make HOMA-IR relatively inadaptable for extensive application in clinical practice. As a surrogate marker of IR, the TyG index, which is simply derived from fasting TG and FBG, has been established and proved to be highly associated with IR evaluated by the gold standard (8). The merits of simplicity, accessibility, inexpensiveness, and insulin-independence make it a more promising indicator for the estimation of IR levels, particularly in daily clinical work and large-scale epidemiological researches.

### Association between TyG index and cardiovascular disease

The significant association of TyG index with the occurrence of cardiovascular disease and recurrent cardiovascular events has been fully investigated and confirmed by certain previous studies. In general population who has never experienced a cardiovascular disease, an elevated TyG index was demonstrated to be observably related to the increased risk of developing CAD, ischemic stroke, and peripheral artery disease (18–21). More importantly, this relationship has been further verified by a recent meta-analysis of eight cohort studies (22), indicating that TyG index plays an important role in early identification of individuals who were at high risk of being subjected to cardiovascular disease. On the other hand, for patients who have experienced cardiovascular disease, a great deal of studies has shown that TyG index is significantly and independently associated with the risk of recurrent cardiovascular events (14, 23, 24), suggesting the great potential of TyG index as a valuable prognostic predictor.

Previous studies have revealed that IR assessed by HOMA-IR is significantly related to coronary plaque vulnerability (25, 26). However, as mentioned above, the insulin-dependence of HOMA-IR restrains it from extensive application in clinical practice. An elevated TyG index has been revealed to be closely related to higher prevalence of unstable carotid plaque identified by ultrasonography (27) and increased coronary plaque volume assessed by coronary computed tomography angiography (28). The association between TyG index and coronary plaque characteristics, confirmation of which can further expose the underlying mechanism mediating the relationship of TyG index with recurrent coronary events at the pathological and anatomical level, however, has not been fully investigated. The present study, which verified the close association of TyG index with non-culprit plaque characteristics and further identified the independent role of TyG index on the prediction of high-risk non-culprit plaques including TCFA and ruptured plaque, fills in the blank of previous studies in this field. It has been also revealed that after being introduced into a baseline model including traditional risk factors, TyG index significantly improved the predictive value for non-culprit TCFA, suggesting that more messages involving the prediction of vulnerable plaques can be offered by TyG index on the basis of traditional risk factors. The results of the present study indicate the great potential of TyG index to be served as a significant surrogate marker of IR providing valuable information on the evaluation of non-culprit plaque features in patients with ACS.

### Potential mechanisms for the relationship of TyG index with cardiovascular disease

As previous study demonstrated, fasting TG and FBG, the determinants of TyG index, mainly reflects the level of IR from adipose tissue and liver respectively, which are the two most significant dimensions for the IR of whole organism (10). The close correlation between TyG index and the hyperinsulinaemic-euglycaemic clamp, the gold standard technique for assessing the IR, has been exactly confirmed by former studies (8, 9), indicating the important role of IR in mediating the association between TyG index and cardiovascular disease. It has been elucidated that IR is closely related to endothelial dysfunction, oxidative stress, proliferation and migration of smooth muscle cells, and activation of inflammatory reaction (6, 7, 29–31), all of which have been recognized as important pathogenesis for the formation of atherosclerosis. Furthermore, TyG index has been also revealed to be correlated to thrombosis imbalance, cardiovascular remodeling, microcirculatory dysregulation, and impaired myocardial perfusion (6, 7, 32), which may be the underlying explanations for the significant predictive value of TyG index for recurrent cardiovascular events.

Additionally, it has been also revealed that TyG index is significantly associated with vascular calcification (33, 34) and arterial stiffness (35), both of which are indicators of atherosclerosis and important for early prediction of cardiovascular disease. Moreover, former studies have also demonstrated that individuals with a higher level of TyG index were often exhibited to be more likely to combine with various traditional risk factors for cardiovascular disease including diabetes (10), prediabetes (11), hypertension (12, 36), renal dysfunction (37), and hyperuricemia (38).

### Therapeutic implications of IR for cardiovascular disease

Previous studies showed that therapeutic lifestyle changes including Mediterranean diet, plenty of exercises, and loss of weight may significantly mitigate the degree of IR, then improve the condition of cardiovascular risk factors and decrease the risk of cardiovascular disease (39–42). As the most evidenced insulin-sensitizing agent, pioglitazone has been verified to have prominent effects on reducing cardiovascular risks in patients with various glucometabolic statuses, despite of with or without baseline cardiovascular disease (43–45). It has been further revealed that the protective effects of pioglitazone on cardiovascular disease may be induced by increased insulin sensitivity and improved IR, rather than decreased blood glucose (46). The results of previous studies indicated that interventions against IR may play an important role in the prevention of cardiovascular disease and recurrent adverse events. Regarding the significant association between TyG index and coronary plaque characteristics having been confirmed by the present study, there is a need for further interventional trials to investigate whether interventions targeting at alleviating the extent of IR assessed by TyG index perform a positive impact on the deferral or even reversion of the progression of coronary plaques.

### Study limitations

Following limitations of the present study should be noted. Firstly, this is a single-center, observational, and cross-sectional study, which makes it hard to identify the causal relationship between TyG index and high-risk plaques. And the relatively smaller sample size may limit the statistical power. Secondly, only patients with ACS and received OCT examinations were enrolled for the current analysis, which may cause selection bias and inextensibility to other population. Thirdly, the hyperinsulinaemic-euglycaemic clamp and HOMA-IR were not accessible in this study, leading to the lack of comparison between TyG index and them in identifying high-risk plaques. Finally, TyG index and OCT-derived plaque characteristics were evaluated only once during hospitalization, further studies are required to dynamically monitor TyG index and plaque characteristics, with the aim to investigate the association between change in TyG index and plaque progression.

## Conclusion

TyG index is significantly and independently associated with TCFA and ruptured plaque identified by OCT examination in patients who were diagnosed with ACS. TyG index, a surrogate marker of IR simply calculated from fasting TG and FBG, can be served as an important and independent risk predictor for high-risk non-culprit coronary plaques in patients who experience an ACS.

## Data availability statement

The original contributions presented in the study are included in the article/Supplementary material, further inquiries can be directed to the corresponding author.

## Ethics statement

Written informed consent was obtained from the individual(s) for the publication of any potentially identifiable images or data included in this article.

## Author contributions

Z-WZ made substantial contributions to study design, data analysis, and manuscript writing. Y-JZ made substantial contributions to study design, intellectual direction, and manuscript revision. CL, QZ, Y-KX, Y-JC, and T-NS made substantial contributions to data collection and interpretation. All authors have read and approved the final manuscript.

## Funding

This work was supported by the grant from National Key Research and Development Program of China (2017YFC0908800), Beijing Municipal Administration of Hospitals Mission plan (SML20180601), Capital's Funds for Health Improvement and Research (CFH2020-2-2063), KM200910025012, and Beijing Municipal Natural Science Foundation (7202041).

## Conflict of interest

The authors declare that the research was conducted in the absence of any commercial or financial relationships that could be construed as a potential conflict of interest.

## Publisher's note

All claims expressed in this article are solely those of the authors and do not necessarily represent those of their affiliated organizations, or those of the publisher, the editors and the reviewers. Any product that may be evaluated in this article, or claim that may be made by its manufacturer, is not guaranteed or endorsed by the publisher.
