# Supplementary material for: Triglyceride-glucose index and non-culprit coronary plaque characteristics assessed by optical coherence tomography in patients following acute coronary syndrome: A cross-sectional study
Source: Front Cardiovasc Med. 2022 Oct 12;9:1019233. doi: 10.3389/fcvm.2022.1019233 (PMC9596751; doi:10.3389/fcvm.2022.1019233)
Supplement: Supplementary file 1 [file Table_1.DOCX]

Table S1. Univariate logistic regression analysis evaluating the possible factors associated with non-culprit TCFA and ruptured plaque

|  | TCFA | | | Ruptured plaque | | |
| --- | --- | --- | --- | --- | --- | --- |
|  | OR | 95% CI | P value | OR | 95% CI | P value |
| Age | 1.025 | 0.990-1.062 | 0.160 | 0.999 | 0.961-1.038 | 0.950 |
| Gender, female | 1.631 | 0.650-4.096 | 0.298 | 0.421 | 0.114-1.553 | 0.194 |
| BMI | 0.993 | 0.873-1.130 | 0.918 | 0.985 | 0.850-1.141 | 0.837 |
| Current smoking | 0.756 | 0.332-1.725 | 0.507 | 0.913 | 0.359-2.325 | 0.849 |
| Hypertension | 0.804 | 0.349-1.851 | 0.607 | 0.296 | 0.112-0.783 | 0.014 |
| Diabetes mellitus | 1.984 | 0.832-4.734 | 0.122 | 0.847 | 0.299-2.402 | 0.755 |
| Previous MI | 2.267 | 0.801-6.411 | 0.123 | 2.375 | 0.776-7.268 | 0.130 |
| Previous PCI | 3.000 | 1.063-8.466 | 0.038 | 0.768 | 0.202-2.930 | 0.700 |
| Diagnosis, STEMI | 0.703 | 0.180-2.745 | 0.613 | 2.941 | 0.856-10.102 | 0.087 |
| TG | 3.260 | 1.850-5.746 | <0.001 | 1.959 | 1.136-3.378 | 0.016 |
| TC | 1.751 | 1.123-2.731 | 0.014 | 0.827 | 0.490-1.395 | 0.475 |
| LDL-C | 1.881 | 1.099-3.221 | 0.021 | 0.923 | 0.502-1.698 | 0.797 |
| HDL-C | 0.223 | 0.039-1.277 | 0.092 | 0.057 | 0.005-0.608 | 0.018 |
| hs-CRP | 1.044 | 0.975-1.118 | 0.218 | 1.029 | 0.956-1.106 | 0.447 |
| Creatinine | 0.993 | 0.964-1.024 | 0.671 | 1.012 | 0.978-1.047 | 0.499 |
| Uric acid | 1.003 | 0.999-1.008 | 0.124 | 1.002 | 0.997-1.007 | 0.423 |
| FBG | 1.512 | 1.033-2.212 | 0.033 | 1.805 | 1.174-2.774 | 0.007 |
| HbA1c | 1.406 | 0.900-2.199 | 0.135 | 1.267 | 0.780-2.057 | 0.338 |

*BMI* body mass index, *MI* myocardial infarction, *PCI* percutaneous coronary intervention, *STEMI* ST-segment elevation myocardial infarction, *TG* triglyceride, *TC* total cholesterol, *LDL-C* low-density lipoprotein cholesterol, *HDL-C* high-density lipoprotein cholesterol, *hs-CRP* high-sensitivity C-reactive protein, *FBG* fasting blood glucose, *HbA1c* glycosylated hemoglobin A1c
